# Tau neuropathology correlates with FDG-PET, but not AV-1451-PET, in progressive supranuclear palsy

**DOI:** 10.1007/s00401-016-1650-1

**Published:** 2016-11-29

**Authors:** Ruben Smith, Michael Schöll, Michael Honer, Christer F. Nilsson, Elisabet Englund, Oskar Hansson

**Affiliations:** 1 Department of Neurology, Skåne University Hospital, 20502 Malmö, Sweden; 2 Clinical Memory Research Unit, Department of Clinical Sciences Malmö, Lund University, Lund, Sweden; 3 Department of Clinical Neuroscience, MedTech West and the University of Gothenburg, Gothenburg, Sweden; 4 Roche Pharmaceutical Research and Early Development, Neuroscience Discovery and Biomarkers, Roche Innovation Center Basel, Basel, Switzerland; 5 Division of Oncology and Pathology, Department of Clinical Sciences Lund, Lund University, Lund, Sweden; 6 Memory Clinic, Skåne University Hospital, 20502 Malmö, Sweden

Radiotracers for tau have recently become available for positron emission tomography (PET) studies in neurodegenerative disorders. The currently most utilized tracer ^18^F-AV-1451 binds to paired helical filaments containing both 3R and 4R tau in Alzheimer’s disease (AD) in vitro [3, 4, 6]. Furthermore, ^18^F-AV-1451 can quantify AD-like tau pathology in *MAPT R406W* mutation carriers in vivo [7]. In corticobasal degeneration, there is a clear binding to 4R tau pathology, but SUVRs are generally lower than in AD [2, 5].

Whether the tracer binds to the straight filament 4R pathology in progressive supranuclear palsy (PSP) is not fully established. Autoradiographic studies [3, 4, 6, 8] have not shown convincing binding of AV-1451 to tau pathology in PSP. We have recently shown increased retention in vivo of ^18^F-AV-1451 in the basal ganglia, but not in the cortex, of patients with PSP [8]. Here, we present data from a 71-year-old male subject, who was diagnosed with PSP in 2011 after a 2-year history of increasing bradykinesia, rigidity, and falls (see Supplementary material for detail). Seven months prior to death the subject was scanned with ^18^F-AV-1451 PET, ^18^F-AV-FDG-PET, and MRI (Fig. 1a–d). ^18^F-AV-1451 PET showed retention in the basal ganglia, but not cortex (Fig. 1a, c). ^18^F-AV-FDG-PET revealed decreased metabolism in the frontal cortex and basal ganglia (Fig. 1b). Voxel-based morphometry showed cortical atrophy, predominantly in the frontal cortex (Fig. 1d). For methodological data, see the supplement. *Post mortem* neuropathological assessment confirmed the diagnosis of PSP, with a marked atrophy of the midbrain and a frontal cortical atrophy. Gallyas silver stain showed dense neurites and stained cell bodies in the basal ganglia, midbrain, and cerebral cortex, as well as cortical tufted astrocytes. Immunohistochemistry showed positivity for 4R tau inclusions, but no 3R tau staining (see supplement for method). Amyloid stains as well as α-synuclein immunohistochemistry were negative (data not shown). Tau-positive (AT8 immunohistochemistry) neurites and cell bodies were quantified using unbiased stereology (see supplement and [7] for methods), and results were correlated with ^18^F-AV-1451 and ^18^F-FDG retention in the same regions (Fig. 1 ). In the frontal, temporal, and parietal cortex and subcortical white matter, there was widespread tau pathology, whereas the occipital lobe and cerebellar grey matter were relatively spared (Fig. 1e–p). The basal ganglia and mesencephalon showed a widespread and intense tau pathology using immunohistochemistry. Comparing the ^18^F-AV-1451 retention to neurite density, there was no correlation between PET signal and the intensity of the pathology (*r*_s_ = 0.23; *p* = 0.29; Fig. 1q). Notably, the highest standardized uptake value ratios (SUVRs) were seen in the globus pallidus, putamen, and substantia nigra, areas with previously reported off-target binding [1, 4, 8]. The cortical and white matter ^18^F-AV-1451 SUVRs were low (<1.2) and showed no association with tau immunohistochemistry. Furthermore, autoradiography of AV-1451 and THK-5351 showed no specific binding to tau pathology (Supplementary Fig. 1). ^18^F-FDG retention in the cerebral cortex, brain stem, and basal ganglia showed a clear inverse correlation to tau neuropathology (*r*_s_ = −0.78; *p* = 0.01; Fig. 1r).

**Fig. 1:**
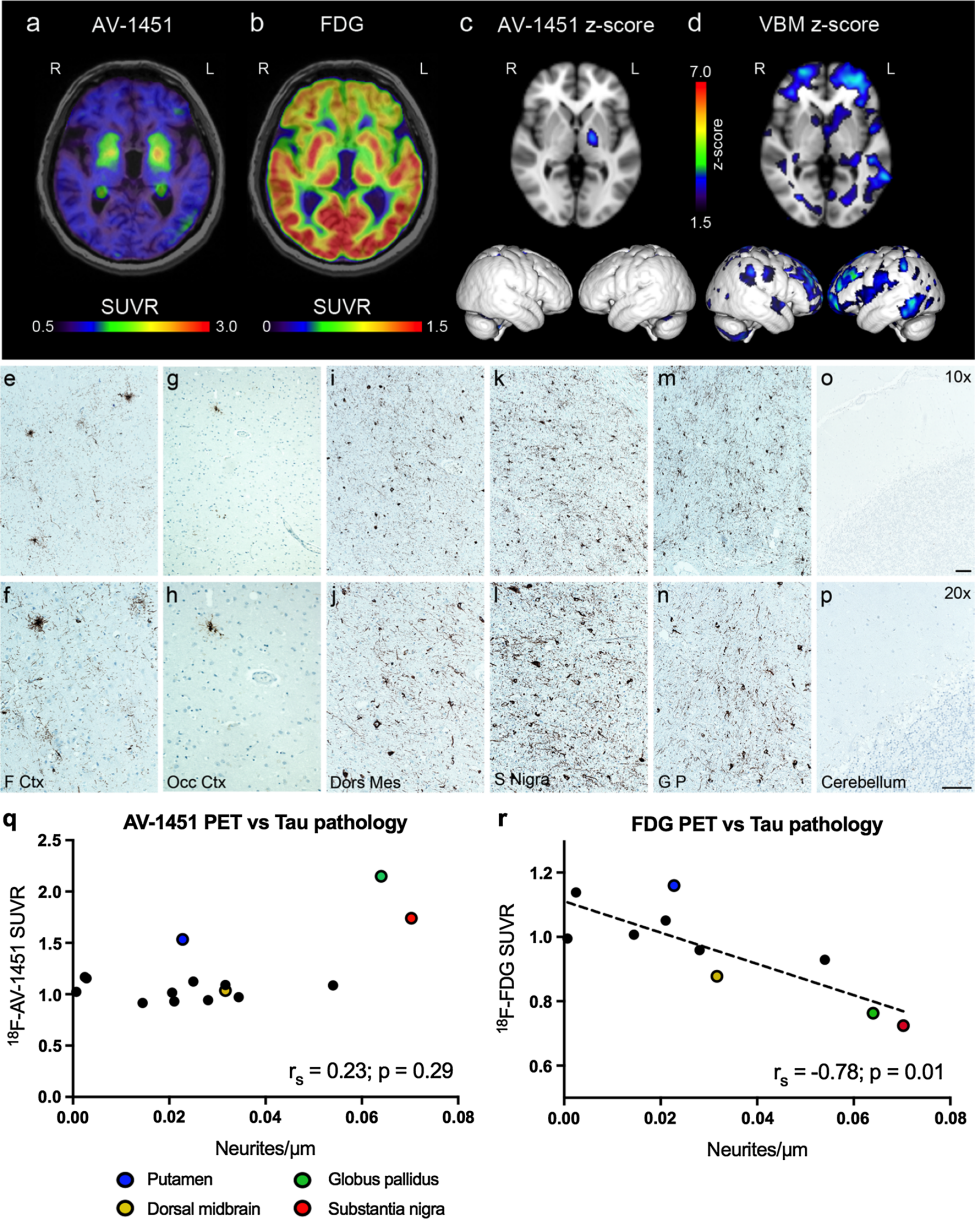
PET retention and neuropathology. **a** SUVR PET image of AV-1451. **b** SUVR PET image of FDG. **c** Transversal and lateral view z-score images of AV-1451 PET signal. **d** Transversal and lateral view z-score of atrophy maps based on inverted voxel-based morphometry. For both z-score images, pixels with values >1.5 (vs controls) are shown. AT8-immunohistochemistry at ×10 (*upper panel*) or ×20 (*lower panel*) magnification of frontal pole cortex (**e**, **f**), occipital cortex (**g**, **h**), dorsal midbrain (**i**, **j**), substantia nigra (**k**, **l**), globus pallidus (**m**, **n**), and cerebellar cortex (**o**, **p**). *Scale bars* 50 µm. **q** ^18^F-AV-1451 SUVRs plotted against the tau-positive neurite density. An annotated version of this figure is available as Supplementary Fig. 2. **r** ^18^F-FDG SUVRs plotted against neurite density

In conclusion, the ^18^F-AV-1451 uptake in cerebral cortex and white matter does not reflect the tau pathology in PSP, possibly due to a less abundant tau pathology compared to AD and a lower affinity of AV-1451 for straight 4R filaments. In the basal ganglia and brain stem, where off-target binding is strong, it is unclear whether the increased ^18^F-AV-1451 reflects true tau pathology, even though the absence of specific autoradiographic binding in these structures argues against this notion. However, an increase in tau pathology is clearly associated with a decrease in ^18^F-FDG-PET.

## Electronic supplementary material

Below is the link to the electronic supplementary material.

Supplementary material 1 (DOCX 175 kb)

Supplementary material 2 (TIFF 13924 kb)

Supplementary material 3 (TIFF 650 kb)
