# Supplementary material for: Tau neuropathology correlates with FDG-PET, but not AV-1451-PET, in progressive supranuclear palsy
Source: Acta Neuropathol. 2016 Nov 29;133(1):149–51. doi: 10.1007/s00401-016-1650-1 (PMC5209394; doi:10.1007/s00401-016-1650-1)
Supplement: Supplementary file 1 — Supplementary material 1 (DOCX 175 kb) [file 401_2016_1650_MOESM1_ESM.docx]

*Supplementary material*

**Tau neuropathology correlates with FDG-PET, but not AV-1451-PET, in Progressive Supranuclear Palsy**

Ruben Smith, Michael Schöll, Michael Honer, Christer F Nilsson, Elisabet Englund and Oskar Hansson.

**Methods**

*Participants*

All procedures conformed to the Declaration of Helsinki and were approved by the Regional Ethical Review Board at Lund University as well as the Swedish Medical Products Agency. Informed written consent was obtained from the patient and controls before inclusion in the study.

The patient had a university education and presented at the age of 66 with a two-year history of progressive balance problems with falls, slowness of thought, speech and writing as well as memory problems. At presentation, examination showed signs of impaired balance, partial vertical gaze palsy and a predominantly right-sided mild bradykinesia and rigidity. Cognitive screening showed MMSE 26/30 and Addenbrooke’s Cognitive Examination 75/100, with problems of attention, delayed recall and verbal fluency. An extensive neuropsychological examination demonstrated a moderate reduction of both verbal and visual episodic memory, verbal fluency, psychomotor speed, attention and planning, while visuospatial function was not affected.

During the subsequent years the patient’s condition worsened gradually. Two years after the baseline assessment, MMSE was unchanged at 28, while behavioural problems such as apathy, lack of interest in relatives and personal hygiene, decreased flexibility and logopenia became more pronounced, with a Frontal Behavioural Inventory score of 24 and Clinical Dementia Rating Scale Sum of Boxes 4.0.

On examination at the time of the PET investigations, the patient had a PSP Rating Scale score of 64. The patient was wheelchair bound, had a pronounced asymmetric bradykinesia and rigidity, dysphagia and hypersalivation. Severe psychomotor slowing and logopenia. The patient died from pneumonia seven months after PET was performed, approximately eight years after symptom onset.

Controls were age-matched, neurologically healthy subjects, recruited from the Swedish Biofinder study (www.biofinder.se).

*Imaging*

*MRI*

The subjects underwent 3.0T MRI-scans on a Siemens Skyra scanner (Siemens Medical Solutions, Erlangen, Germany). Acquired sequences were fluid-attenuated inversion recovery (FLAIR) and T1-weighted magnetization-prepared rapid gradient echo (t1-mprage).

*PET*

The radiosynthesis has been described in detail previously[1]. The radioligand was administered as an intravenous bolus injection of 370 MBq of ^18^F-AV1451. Emission data was acquired in list-mode over 80-140 min post injection using a GE Discovery 690 PET/CT system (GE Healthcare, Milwaukee, USA). Reconstruction parameters have been described in detail previously[1]. In brief the list -mode data was motion corrected and binned into 5 min time frames. A low-dose computed tomography (CT) scan was performed prior to the PET scan for attenuation correction. ^18^F-FDG scans were acquired as a static 20 min scan 60 min after injection of 200 MBq ^18^F-FDG.

PET data was analyzed further using Pmod version 3.705 (Pmod technologies llc., Zurich, Switzerland). AV-1451 PET images were motion-corrected and averaged using the View Tool. Rigid body affine coregistration of PET and MRI scans, as well as MRI grey-white matter segmentation, spatial normalization, and transformation of template ROIs to PET space were performed using the Neuro Tool. The Automatic Anatomic Labelling (AAL) template was used. Cortical ROIs were masked using a grey matter probability map with a threshold set at 0.75 and transformed to PET space using the affine transformation obtained from MR-to-PET coregistration. Cerebellar grey matter, not including the dentate nucleus and vermis, was used as a reference region. SUVR-images were created using the cerebellar reference region. Using the T1-MPRAGE MRI-image cortical, basal ganglia, midbrain and white matter areas were hand-drawn in the same regions where samples for neuropathology were taken. The AV-1451 and FDG SUVR-images were coregistered onto the T1-MPRAGE image and the SUVR-values in the regions of interest were calculated.

*Voxelbased morphometry and z-scores maps*

Voxel-based morphometry (VBM) as implemented in SPM12 (Wellcome Department of Cognitive Neurology, London, UK; [http://www.fil.ion.ucl.ac.uk/spm](https://email.ki.se/owa/redir.aspx?SURL=6njNG5OnL9TygKIsNXJ_bxA-T8dkViAacqXqhzRBUMRUxywLO1rTCGgAdAB0AHAAOgAvAC8AdwB3AHcALgBmAGkAbAAuAGkAbwBuAC4AdQBjAGwALgBhAGMALgB1AGsALwBzAHAAbQA.&URL=http%3a%2f%2fwww.fil.ion.ucl.ac.uk%2fspm)) was used to evaluate GM intensity differences as a measure of GM atrophy. In preparation, all individual T1-weighted MR images were segmented into tissue classes, and the GM segmentations subsequently warped into a common MNI152 standard space (using a cohort-specific template created with the DARTEL toolbox and Jacobian-scaling to estimate GM intensity). The resulting maps were smoothed with an 8 mm full-width at half maximum (FWHM) Gaussian kernel.

Using FSL (http://fsl.fmrib.ox.ac.uk), we then created an average and standard deviation image based on the control group’s VBM and AV-1451 images that had been warped into a common MNI152 standard space employing their coregistered MRI scans and the above-mentioned cohort-specific template. Subsequently, we created a z-score map for the inverted VBM image (less GM intensity as a measure for GM atrophy) and the AV-1451 image for the case described in this article. Z-score images were generated using Mango (version 4.0; Research Imaging Institute, UTHSCSA, USA).

*Neuropathology*

The post mortem interval was 48 hours. The brain was fixed in 6 % formaldehyde solution, cut in bihemispheric coronal sections and embedded in paraffin. Neuropathological diagnostic analysis was performed on coronal sections including the globus pallidus/putamen, the hippocampus, the inferior temporal gyrus, the frontal pole and the dorsolateral frontal gyri, the parietal and occipital cortex, plus sections covering the midbrain and the cerebellum. Paraffin-embedded sections were stained with Luxol fast blue myelin stain (LFB), hematoxylin and eosin (H&E), and Gallyas silver stain. Immunohistochemistry was performed on 5 μm-thick tissue sections using antibodies for phosphorylated tau (AT8, 1:200, DAKO), 4R tau (Anti-4R-tau, rabbit polyclonal antibody, 1:3000, CosmoBio), 3R tau (RD3, 8E6/C11, 1:1000, Millipore) and α-synuclein (clone LB509, 1:600, Zymed laboratories). For immunohistochemistry, all sections were microwave pre-treated in 10 mM citrate buffer at pH 6.0 and 100 degrees C for 10 minutes, in order to achieve antigen retrieval. An automated immunostainer (Dako Autostainer Plus, DAKO Sweden AB) was used for the staining procedure using DAKO ChemMate Kit Peroxidase/3,3′-diaminobenzidine. α-synuclein pathology was assessed immunohistochemically and Congo Red staining was applied for the detection of amyloid on a large hemispheric section.

*Stereology*

To assess the amount of tau-neuropathology stereological systematic random sampling (Stereo Investigator, MBF Bioscience) was used. The number of tau-positive neurites/µm, as well as the number of tau-positive cells/mm^2^ were estimated. The analysis was performed in the cortex and underlying white matter of AT8-stained cortical sections and in basal ganglia and midbrain sections.

*In vitro autoradiography*

Fresh frozen frontal cortex tissue blocks (superior frontal sulcus) from 3 PSP patients were obtained from the Neurodegenerative Disease Brain Bank (San Francisco, CA, USA). A mid-temporal gyrus block with high tau load from a late stage AD patient (Braak VI) was used as a positive control and was purchased from the Banner Sun Health Research Institute (Sun City, AZ, USA). Ten µm thick sections of the brain tissue blocks were generated with a cryostat (Leica CM3050) at -17 °C chamber temperature and -15 °C object temperature. Sections were transferred to Histobond+ microscope slides (Marienfeld Laboratory Glasware). After drying for 3 h at room temperature the sections were stored at -20 °C.

AV-1451 was tritiated at Roche with a specific activity of 29 Ci/mmol and radiochemical purity better than 98%. THK5351 was also tritiated at Roche with a specific activity of 45 Ci/mmol, and radiochemical purity greater than 98%. The brain sections were incubated with 10nM radioligand in 50 mM Tris buffer, pH 7.4 at room temperature for 30 min. After washing 3x 10 min at 4 °C in 50 mM Tris buffer, pH 7.4 and 3 quick dips in H_2_O dist. at 4 °C the sections were dried at 4 °C for 3 h. The sections were placed in a FujiFilm Cassette (BAS 2025), exposed with a FujiFilm Imaging Plates (BAS-IP TR 2025) for five days and afterwards scanned with a FujiFilm IP reader (BAS-5000) with a resolution of 25 µm per pixel. The autoradiograms were visualized with the software MCID analysis (version 7.0, Imaging Research Inc.). The presence of tau aggregates was confirmed on adjacent sections using the tau-specific antibody AT-8 conjugated with Alexa555 (5 µg/mL).

*Statistics*

Spearman correlations were used for assessing statistical significance using Graph Pad Prism 7 for Macintosh. Spearman rho (r_s_) values are described along with p-values. P-values < 0.05 were considered significant.

**References**

1 Smith R, Schain M, Nilsson C et al (2016) Increased basal ganglia binding of 18 F-AV-1451 in patients with progressive supranuclear palsy. Mov Disord: Doi 10.1002/mds.26813
