# Supplementary figures and images for: Tau neuropathology correlates with FDG-PET, but not AV-1451-PET, in progressive supranuclear palsy
Source: Acta Neuropathol. 2016 Nov 29;133(1):149–51. doi: 10.1007/s00401-016-1650-1 (PMC5209394; doi:10.1007/s00401-016-1650-1)

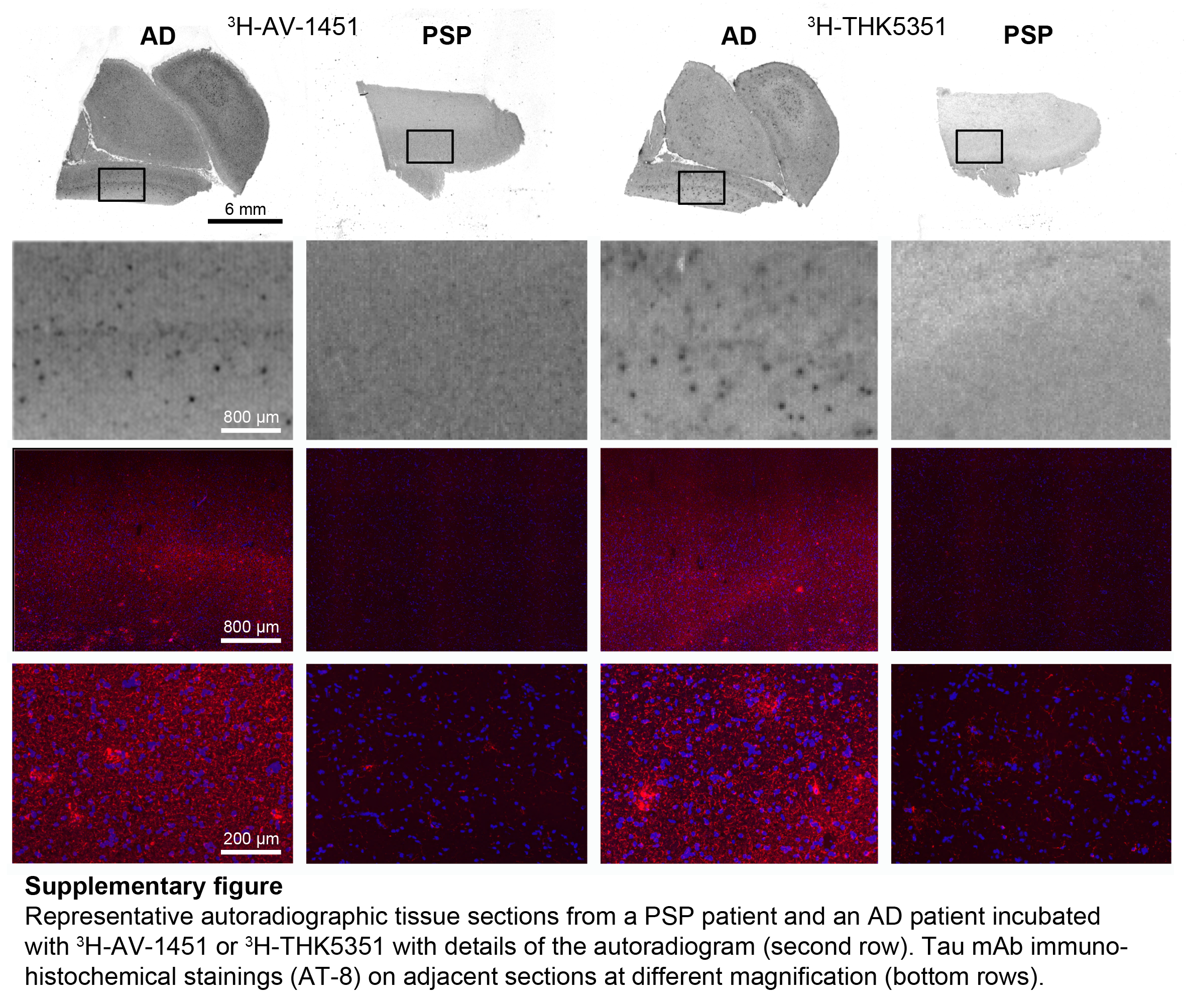

Supplement: Supplementary file 2 — Supplementary material 2 (TIFF 13924 kb) [file 401_2016_1650_MOESM2_ESM.tif]

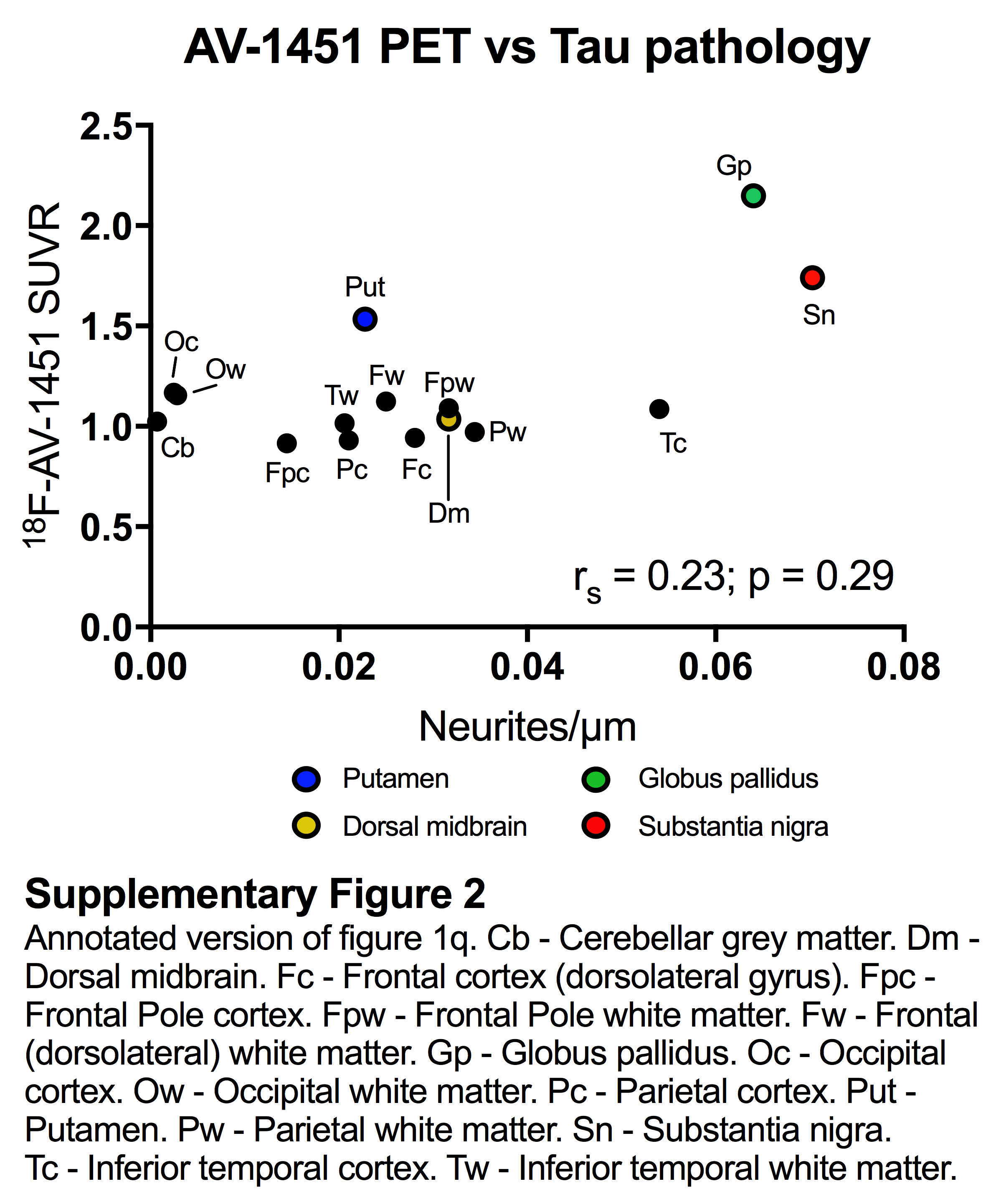

Supplement: Supplementary file 3 — Supplementary material 3 (TIFF 650 kb) [file 401_2016_1650_MOESM3_ESM.tiff]
